# Uncoupling the Functions of CALM in VAMP Sorting and Clathrin-Coated Pit Formation

**DOI:** 10.1371/journal.pone.0064514

**Published:** 2013-05-31

**Authors:** Daniela A. Sahlender, Patrycja Kozik, Sharon E. Miller, Andrew A. Peden, Margaret S. Robinson

**Affiliations:** Cambridge Institute for Medical Research, University of Cambridge, Cambridge, United Kingdom; NHLBI, NIH, United States of America

## Abstract

CALM (clathrin assembly lymphoid myeloid leukemia protein) is a cargo-selective adaptor for the post-Golgi R-SNAREs VAMPs 2, 3, and 8, and it also regulates the size of clathrin-coated pits and vesicles at the plasma membrane. The present study has two objectives: to determine whether CALM can sort additional VAMPs, and to investigate whether VAMP sorting contributes to CALM-dependent vesicle size regulation. Using a flow cytometry-based endocytosis efficiency assay, we demonstrate that CALM is also able to sort VAMPs 4 and 7, even though they have sorting signals for other clathrin adaptors. CALM homologues are present in nearly every eukaryote, suggesting that the CALM family may have evolved as adaptors for retrieving all post-Golgi VAMPs from the plasma membrane. Using a knockdown/rescue system, we show that wild-type CALM restores normal VAMP sorting in CALM-depleted cells, but that two non-VAMP-binding mutants do not. However, when we assayed the effect of CALM depletion on coated pit morphology, using a fluorescence microscopy-based assay, we found that the two mutants were as effective as wild-type CALM. Thus, we can uncouple the sorting function of CALM from its structural role.

## Introduction

Proteins belonging to the CALM/AP180 family are found in nearly all eukaryotes, and are major components of the coats on endocytic clathrin-coated vesicles (CCVs). Mammals have two proteins belonging to the CALM/AP180 family, the ubiquitously expressed CALM and the neuronal-specific AP180, while most organisms have only a single family member. The first insights into the functions of this family came from a study on UNC-11, the CALM/AP180 protein in C. elegans. Two defects were found in the neurons of *unc-11* mutants: they contained abnormally large synaptic vesicles, and the R-SNARE synaptobrevin, which is normally found almost exclusively in synaptic vesicles, was mislocalised to the plasma membrane [1]. SNAREs are essential components of transport vesicles, required for the vesicles to fuse with their target membrane, so this observation provided an important clue about how synaptobrevin might be recognized as vesicle cargo.

Subsequent studies showed that members of the CALM/AP180 family are involved in both vesicle size control and R-SNARE sorting not only in neurons, but also in other types of cells. For instance, CALM depletion in HeLa cells causes the cells to form larger and more irregular clathrin-coated pits at the plasma membrane, although the pits are still functional for clathrin-mediated endocytosis [2]. Knocking out the two redundant family members in Saccharomyces cerevisiae, Yap1801 and Yap1802, profoundly affects the internalization of the R-SNARE Snc1, without affecting the internalization of other CCV cargo proteins [3]; and knocking down CALM in human embryonic kidney cells causes transiently transfected VAMP2, another R-SNARE, to accumulate on the plasma membrane [4]. Together, these studies suggested that CALM might be an adaptor for certain types of R-SNAREs, even though for many years no physical interactions were reported.

In 2011, two papers were published showing that CALM binds directly to VAMPs 2, 3, and 8, the closest mammalian homologues of worm synaptobrevin and yeast Snc1 [5] [6]. Interestingly, the VAMPs interact with CALM via their SNARE domains, the same domains that are used to form four-helical bundle with other SNAREs to drive membrane fusion [7]. This is in contrast to most other CCV cargo proteins, which bind via short linear motifs [8]. Two other SNAREs, vti1b and VAMP7, have also been shown to bind to their adaptors (epsinR and Hrb, respectively) via folded domains [9] [10] [11]; however, these folded domains are N-terminal to their SNARE domains and are missing in VAMPs 2, 3, and 8, worm synaptobrevin, and yeast Snc1, all of which belong to the brevin family, which lacks folded N-terminal domains.

The ability of CALM to sort SNAREs helps to explain a number of observations. The uncoordinated phenotype of *unc-11* worms is likely to be due to the decreased levels of synaptobrevin in their synaptic vesicles, compromising the vesicles’ ability to fuse with the plasma membrane. Similarly, Drosophila with P-element insertions into their CALM/AP180 gene, LAP, are uncoordinated and sluggish, and usually die as embryos [12]. More recently, genome-wide association studies have implicated CALM variants in Alzheimer’s disease [13], which again may be related to changes in the localisation of SNAREs, because even slight perturbations in the trafficking of the Amyloid Precursor Protein, its binding partners (e.g., SorLA), and/or its proteases can all lead to increases in the production of amyloidogenic peptides [14]. But what is less clear is whether the altered size of clathrin-coated structures in CALM-depleted cells is caused by the missorting of SNAREs, or whether it reflects a different function of CALM.

Here we investigate two questions. First, can CALM sort other post-Golgi SNAREs in addition to the brevins VAMPs 2, 3, and 8? And second, in CALM-depleted cells, is the clathrin-coated pit (CCP) size phenotype a result of the SNARE missorting phenotype?

## Materials and Methods

### Constructs

Most of the HA-tagged VAMP constructs have already been described [6] [15]. The Δlongin VAMP7 construct was generated by PCR, omitting the first 102 residues, and also cloned into the pLIXIN-HA vector. The L16PV23P, K24AM27A, and L44PL51P mutations were made using a QuikChange mutagenesis kit (Stratagene). Human CALM cDNA was amplified by PCR from IMAGE clone 5537605 and made resistant to siRNA by introducing four silent mutations. A myc tag was inserted into a non-conserved region (position 1308 bp), and the construct was cloned into the retroviral vector pIRES-PBMN-1 for low to moderate expression. The S219L and M244K mutations were introduced using QuikChange mutagenesis [6].

### Transfections

For expression of HA-tagged VAMP constructs, HeLa M cells [16] were infected with retrovirus using the Phoenix system, and cell lines were selected with 0.5 mg/ml G418, as previously described [15]. For coexpression of VAMP8-HA and a CALM construct, VAMP8-HA-expressing cells were infected with retrovirus containing a wild-type or mutant CALM cDNA, and cell lines were selected with 0.17 mg/ml hygromycin B plus 0.5 mg/ml G418. At least five different clonal cell lines were generated for each construct.

siRNA knockdowns were carried out using 20 nM siRNA (Dharmacon) with Oligofectamine (Invitrogen) as the transfection reagent, as previously described [17]. Several individual siRNAs were tested for CALM knockdown efficiency and two were chosen for subsequent experiments: oligo 2 (5′-GAAATGGAACCACTAAGAA) and oligo 5 (5′-ACAGTTGGCAGACAGTTTA).

### Immunofluorescence and Western Blotting

Cells were prepared for immunofluorescence either by fixation with paraformaldehyde followed by permeabilization with Triton, or by fixation/permeabilization with methanol at −20°C, and antibody labeling was carried out as previously described [17]. Images were acquired using a Zeiss Axiophot fluorescence microscope equipped with a CCD camera (Princeton Instruments), and photographs were recorded using IP Laboratories software and then moved into Photoshop. SDS-PAGE and Western blotting were performed as described by Gordon et al. [15].

Primary antibodies used in this study included an in-house affinity-purified rabbit antiserum against clathrin heavy chain [18], a commercial goat polyclonal antibody against CALM (Santa Cruz), commercial rabbit polyclonal antibodies against Dab2 (Santa Cruz) and actin (Sigma), and commercial mouse monoclonal antibodies against the HA epitope (Covance), the myc eptiope (Millipore), syntaxin 4, AP-2 α, and AP-2 µ2 (all from BD). Secondary antibodies for immunofluorescence were purchased from Invitrogen. Western blots were visualized by enhanced chemiluminescence (GE Healthcare).

For cycloheximide experiments, VAMP8-HA wt (clone 6), VAMP8-HA L16PV23P (clone 3), VAMP8-HA L44PL51P (clone 5), and VAMP8-HA K24AM27A (clone 3) were treated with 100 ug/ml cycloheximide (Sigma) for 2 or 4 hrs, and cell lysates were analysed by Western blotting as described above.

For pulldowns, recombinant proteins were expressed in BL21(DE3)/pLysS cells and purified as described by Miller et al. [6]. To test the binding of myc-tagged CALM to GST-tagged VAMP7, the proteins were incubated overnight at 4°C with constant agitation, and the pulldown was analysed by SDS-PAGE and Western blotting as described by Miller et al. [6].

### Endocytosis Efficiency Assays

To quantify the endocytosis efficiency HA-tagged VAMPs, a modification of the method first described by Kozik et al. [19] was used. The cells were trypsinized, washed, and incubated for 40 minutes in medium containing anti-HA that had been directly conjugated to AlexaFluor 488, using a protein labeling kit (Invitrogen). This incubation was carried out at 37°C to allow endocytosis to occur. The cells were then placed on ice, washed, and incubated on ice for 30 min in 1% BSA in PBS (PBS-BSA), containing anti-AlexaFluor 488 (Invitrogen) to quench green fluorescence on the cell surface, and Cy5-conjugated goat anti-mouse IgG (Jackson Immuno Research Laboratories) to label the surface antibody in red. Cells were washed again and resuspended in PBS-BSA containing 7-AAD (7-amino-actinomycin D, a viability stain) (Invitrogen) so that dead cells could be excluded. The samples were analysed on a BD FACSCalibur. For each experiment, 10,000 cells were gated for forward/side scatter and 7-AAD exclusion. The mean fluorescence for each sample was calculated using FlowJo software (Tree Star, Inc., Ashland, OR, USA). The ratio of green to red fluorescence was used as a measure of endocytosis efficiency. For the experiment in which only the surface levels of HA-tagged constructs were quantified, the cells were labeled with anti-HA as previously described [15].

### Electron Microscopy

HeLa M cells were depleted of CALM by siRNA knockdown with oligo 5 as described above, fixed with 2.5% GA/2% PFA in 0.1 M sodium cacodylate buffer and processed for resin embedding as described by Hirst et al. [20]. Ultrathin sections were collected onto EM grids and stained with uranyl acetate and Reynolds lead citrate. The sections were observed in a transmission electron microscope (model CM 100; Philips) at 80 kV.

### Automated Fluorescence Microscopy

Four clones of each of the following cell lines: VAMP8-HA wt, VAMP8-HA+CALM-myc wild type, VAMP8-HA+CALM-myc L219S, or VAMP8-HA+CALM-myc M244K, were transfected with oligo 05 to deplete endogenous CALM, and then seeded into 96-well plates 48 hr after transfecting the siRNA. Each clone was plated out in triplicates at a density of 7000 cells per well. After a further 24 hr, cells were fixed in ice-cold methanol for 5 minutes, washed with PBS, incubated in 1% BSA/PBS containing 0.1% Triton X-100 for 10 minutes, and labeled with anti-Dab2 for 45 min. After washing, the cells were labeled with Alexa488-labeled donkey-anti rabbit IgG for 30 minutes, followed by an incubation with whole cell stain (WCS) (Thermo Fisher) for 30 minutes. FLOW-CHECK beads from the APC (675/633) set up kit (Beckman Coulter, Cat. No. 6607120) were used as a focusing reference, as described by Kozik et al. [21]. The images were acquired on the Arrayscan platform (Cellomics, ThermoFisher) with a 40× objective lens using the Spot Detector bioapplication. The Cy5 channel was used to determine a focus plane, and the images of Dab2 were acquired with a −3.5 µm offset. The total intensity per spot was used for analysis; triplicate values for each clone were averaged for each experiment. The data shown are an average of three independent experiments.

## Results

### Endocytosis Efficiency Assay

**Figure 1:**
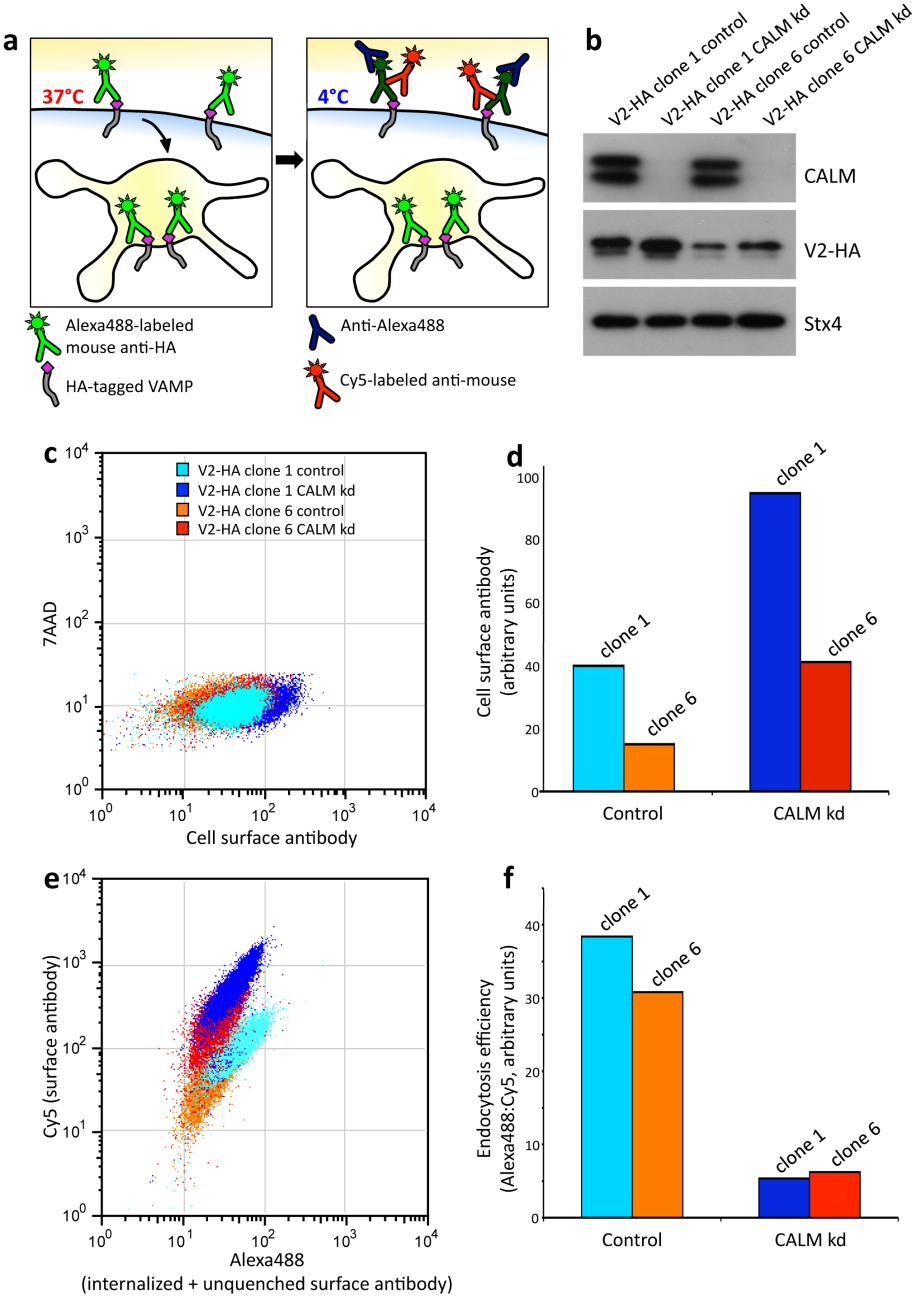
Endocytosis efficiency assay. a, Schematic diagram of the endocytosis efficiency assay. Cells are allowed to internalize an AlexaFluor 488-labeled mouse antibody against the HA tag for 40 minutes at 37°C. They are then cooled to 4°C to stop endocytosis, and an antibody against AlexaFluor 488 is added to quench green fluorescence on the cell surface but not in endosomes, together with a Cy5-labeled anti-mouse antibody to add a red fluorescent tag to the surface antibody. When the cells are analysed by flow cytometry, the green-to-red ratio gives a measure of endocytosis efficiency. b, Western blot of homogenates of control and siRNA-treated cells, probed for CALM, HA-tagged VAMP2 (V2-HA), and (as a loading control) syntaxin 4 (Stx4). Clone 1 expresses more VAMP2-HA than clone 6. The doublets in the CALM lanes are most likely due to alternative splicing [23] [2]. c, Scatter plot showing surface expression of VAMP2-HA, quantified by flow cytometry, for the four different cell populations. d, Bar graph showing that surface expression is dependent on total expression: although the CALM knockdown increases the surface expression of VAMP2-HA about 2.5-fold in both clone 1 and clone 6, the amount of VAMP2-HA on the cell surface in clone 6 after CALM knockdown is similar to the amount of VAMP2-HA on the cell surface in clone 1 under control conditions. e, Scatter plot showing AlexaFluor 488 fluorescence, representing internalized and unquenched surface antibody, on the X axis, and Cy5 fluorescence, representing surface antibody only, on the Y axis. Although clone 1 expresses the construct at higher levels than clone 6, the slopes show that the ratio of AlexaFluor 488 to Cy5 is similar for the two cell lines, both under control conditions and after CALM knockdown. f, Bar graph showing endocytosis efficiency as a ratio of AlexaFluor 488 to Cy5. The values are similar in the two cell lines.

To monitor R-SNARE sorting in different cell lines under different conditions, we needed an assay that would not be affected by variations in expression level. To this end, we modified an endocytosis efficiency assay that we recently developed [19], which is shown diagrammatically in Figure 1a. The assay makes use of clonal cell lines that express low levels [6] of an R-SNARE (VAMP 2, 3, 4, 7, or 8) with a C-terminal (i.e. lumenal/extracellular) double hemagglutinin (HA) tag preceded by a short linker [6] [15]. The cells are allowed to internalize an AlexaFluor 488-labeled mouse antibody against the HA tag for 40 minutes at 37°C. They are then cooled to 4°C to stop further endocytosis, and an antibody against AlexaFluor 488 is added to quench green fluorescence on the cell surface but not in endosomes, together with a Cy5-labeled anti-mouse antibody to add a red fluorescent tag to the surface antibody. When the cells are analysed by flow cytometry, the green-to-red ratio gives a measure of endocytosis efficiency. One of the advantages of the endocytosis efficiency assay is that it is not affected by the expression level of the HA-tagged VAMP, which can vary from one cell line to another, and which can also change when a component of the trafficking machinery is depleted (e.g., there is a small but reproducible increase in the amount of HA-tagged VAMP2 when CALM is knocked down) (Figure 1b). This is in contrast to surface binding assays, which are highly dependent upon expression levels (Figure 1 c–f).

**Figure 2:**
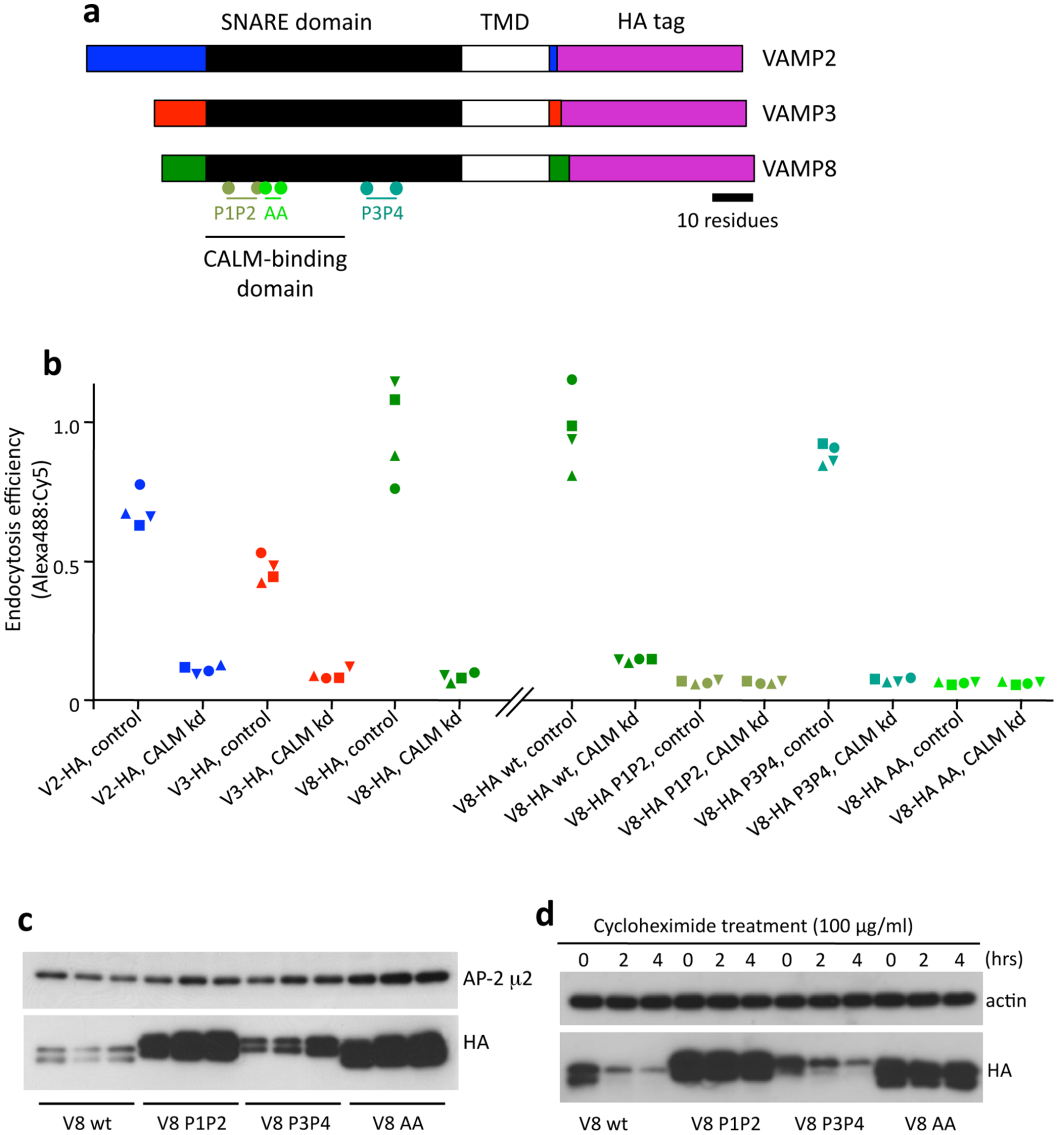
Endocytosis efficiency of VAMPs 2, 3, and 8. a, VAMPs 2, 3, and 8 have a similar domain structure, consisting of a short, apparently unstructured N-terminal domain, a SNARE domain, a transmembrane domain (TMD), and a very short lumenal/extracellular domain, to which a double HA (hemagglutinin) tag was added when making the constructs. The CALM-binding domain and the positions of the residues that were mutated are indicated for VAMP8. b, The FACS-based assay described in Figure 1 shows that endocytosis efficiency is highest for VAMP8, and is strongly reduced when CALM is depleted. The VAMP8 P3P4 mutant, which is not impaired in CALM binding, is endocytosed as efficiently as the wild-type construct, and both are strongly affected by CALM knockdown. In contrast, the P1P2 and AA mutants, which are unable to bind CALM, are poorly endocytosed, and the CALM knockdown has no effect on the endocytosis efficiency of these constructs. c, Western blot showing steady state levels of wild-type and mutant VAMP8 in three different cell lines for each construct. Cell lysates were adjusted for equal protein concentration. Although there is some variability from one line to another, the two non-CALM-binding mutants are always expressed at much higher levels than wild-type VAMP8 or the P3P4 mutant. d, The differences in expression are due to differences in degradation, not in synthesis. When de novo protein synthesis was blocked with cycloheximide, the levels of the two CALM-binding constructs decreased rapidly over four hours, while the levels of the two non-CALM-binding mutants were not appreciably affected.

Proof of principle for this assay is shown in Figure 2. We have previously investigated the sorting of VAMPs 2, 3, and 8, all of which bind CALM (Figure 2a), and have found that all three of these VAMPs are internalised in a CALM-dependent manner. VAMP8 has been shown to bind CALM with the highest affinity (K_D_ ∼18 µm), followed by VAMP2 and VAMP3 (K_D_ ∼43 µm and ∼46 µm respectively) [6]. When we assayed the endocytosis efficiency of HA-tagged VAMPs, using four different cell lines for each construct, we found that differences in affinity could be correlated with differences in endocytosis efficiency (Figure 2b). In every case, endocytosis efficiency went down to baseline levels when we depleted CALM with siRNA.

Next, we investigated the endocytosis efficiency of three different VAMP8 mutants. Two of these mutations, L16PV23P (P1P2) and L44PL51P (P3P4), disrupt the α-helical SNARE domain by changing hydrophobic residues to prolines [15]. The other mutation, K24AM27A (AA), changes two other conserved residues in the SNARE domain to alanines. Only the P1P2 and AA mutations affect the CALM-binding domain, and we have previously shown that both of these mutants accumulate on the cell surface [6]. Figure 2b demonstrates that the P3P4 mutant is still efficiently endocytosed, in a CALM-dependent manner, while the P1P2 and AA mutants are not. The endocytosis efficiency assay was particularly useful for analysis of the various mutants, because the two mutants that fail to be endocytosed were consistently expressed at higher levels, due to reduced rates of degradation (Figure 2 c–d).

### VAMPs 4 and 7

**Figure 3:**
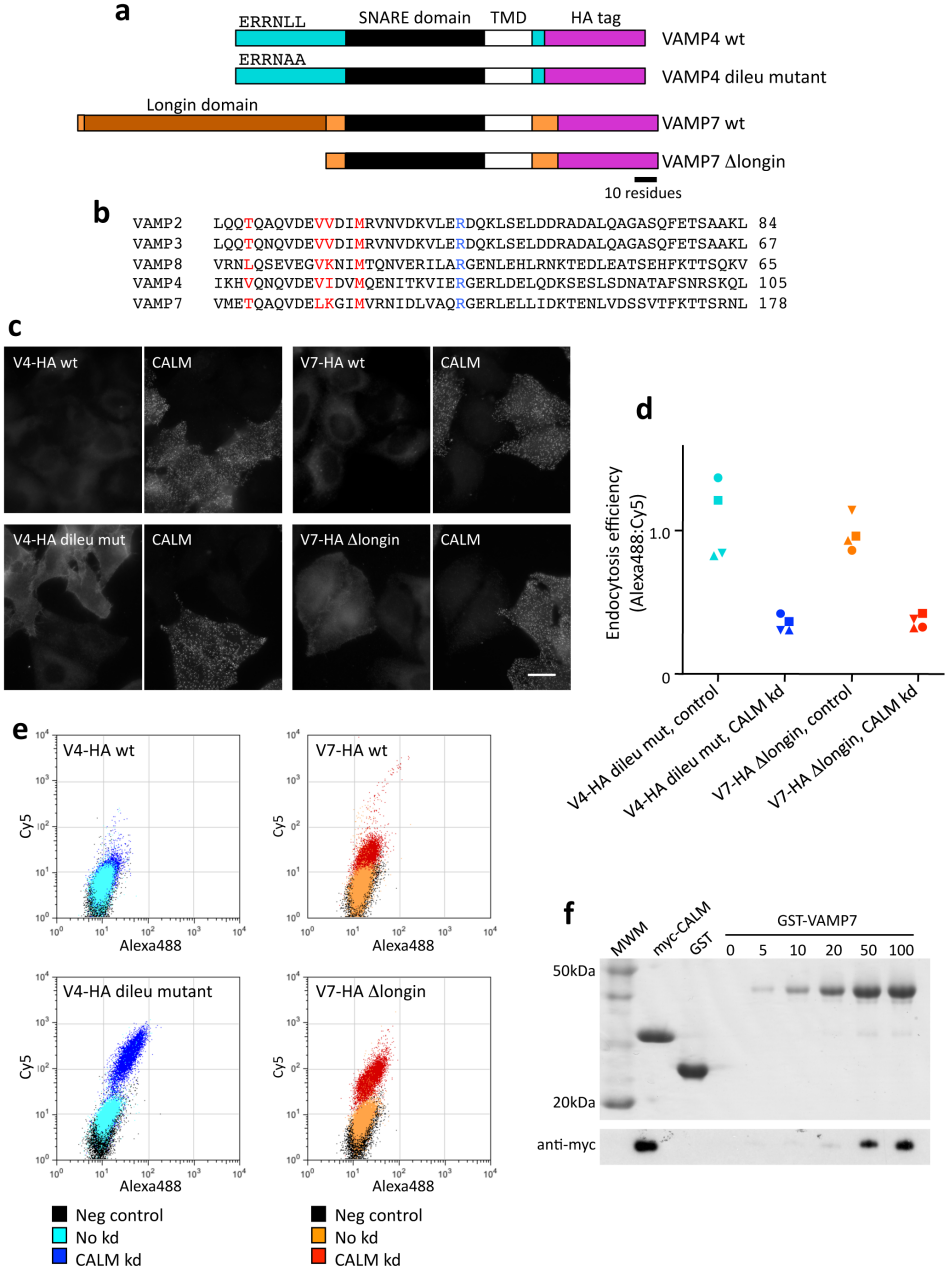
Sorting of VAMPs 4 and 7. a, Schematic diagrams of the wild-type and mutant constructs. b, Alignment of the SNARE domains of the five VAMPs. The residues that were targeted when making the non-VAMP-binding mutants are in red; the key arginine residue found in all R-SNAREs is in blue. c, Effect of CALM knockdown on surface expression of HA-tagged VAMP4 and VAMP7. Control and siRNA-treated cells were mixed together, fixed without permeabilization and labeled with anti-HA, then permeabilized and labeled with anti-CALM. Knocking down CALM increases the surface expression of the mutant VAMPs but does not have any obvious effect on the wild-type VAMPs. Scale bar: 20 µm. d, Endocytosis efficiency of the mutant VAMP4 and VAMP7 constructs. In both cases, CALM knockdown decreases the endocytosis efficiency. The values were normalized to 1 for the VAMP7 control. e, Scatter plots showing AlexaFluor 488 fluorescence, representing internalized and unquenched surface antibody, on the X axis, and Cy5 fluorescence, representing surface antibody only, on the Y axis. The negative control consists of cells that were not exposed to either of the two fluorescent antibodies. There is very little wild-type VAMP4 or VAMP7 on the cell surface, and the CALM knockdown has a negligible effect on wild-type VAMP4, although it does increase the surface expression of wild-type VAMP7. There is more of the two mutant constructs on the cell surface when compared with wild-type, and in both cases the CALM knockdown strongly increases surface expression. f, Pulldown assay with GST-VAMP7. Purified recombinant myc-tagged CALM_ANTH_ domain and GST-VAMP7 (0, 5, 10, 50, or 100 nmoles) were mixed and captured using glutathione-Sepharose. The top panel shows the Coomassie blue-stained gel and the lower panel shows a Western blot of the same gel probed with anti-myc. Even with its longin domain, the VAMP7 construct binds CALM_ANTH_ at high concentrations.

There are two other post-Golgi VAMPs that have an intracellular distribution, VAMP4 and VAMP7 (15) (Figure 3a). Unlike VAMPs 2, 3, and 8, VAMPs 4 and 7 are able to bind to other clathrin adaptors. VAMP4 has a classical dileucine motif, ERRNLL, and can bind adaptor protein (AP) complexes [22], while VAMP7 binds the alternative adaptor Hrb via its N-terminal longin domain [10] [11]. However, they share residues important for CALM binding with VAMPs 2, 3, and 8 (Figure 3b).

To find out whether VAMPs 4 and 7 might also be sorted by CALM, we generated stable HeLa cell lines expressing HA-tagged versions of both proteins, and then carried out CALM knockdowns using siRNA. When we mixed together control and siRNA-treated cells and labeled for construct on the cell surface with an antibody against the tag, we did not see any obvious difference in the CALM-depleted cells (Figure 3c, upper panels). This is in contrast to VAMPs 2, 3, and 8, where the CALM-depleted cells are easy to identify because of increased surface expression of the tagged constructs [6].

One reason for the insensitivity of VAMPs 4 and 7 to CALM depletion may be that they use their dileucine motif and longin domain as internalization signals. Thus, we mutated the two leucines in VAMP4 to alanines, and we deleted the longin domain from VAMP7. We then made cell lines expressing these mutants and repeated the experiment. The CALM knockdown could now be seen to cause both the VAMP4 dileucine mutant and the VAMP7 Δlongin mutant to relocate to the plasma membrane (Figure 3c, lower panels).

The CALM knockdown phenotype was further investigated by carrying out the endocytosis efficiency assay. It was difficult to quantify the fluorescence of wild-type VAMPs 4 and 7, because so little of the protein was on the cell surface at steady state that the labeled cells were virtually indistinguishable from negative control cells, which had not been exposed to the primary antibody. However, we saw a clear effect of the CALM knockdown in cells expressing both the VAMP4 mutant and the VAMP7 mutant (Figure 3d). In addition, the raw data provided information about the behavior of the wild-type HA-tagged VAMPs. Figure 3e shows dot plots of representative cell lines expressing wild-type and mutant VAMP4 and VAMP7. In the case of wild-type VAMP4, the CALM knockdown had little or no effect. The VAMP4 dileucine mutant was somewhat elevated on the plasma membrane when compared with wild-type VAMP4, and the CALM knockdown had a strong effect, consistent with the immunofluorescence results. There was also very little wild-type VAMP7 on the plasma membrane. However, in this case there was increased surface expression of the wild-type VAMP7 when CALM was depleted, even though there was not enough to be detected by immunofluorescence. The Δlongin VAMP7 showed the expected phenotype: it was somewhat elevated on the plasma membrane when compared with wild-type, and it was strongly affected by the CALM knockdown.

The effect of the CALM knockdown on wild-type VAMP7 was surprising, because we had assumed that the longin domain would occlude the binding site on VAMP7 for CALM. The interaction between VAMP7 and CALM was further investigated in vitro using purified proteins. Although we were unable to detect any binding between CALM and VAMP7 by ITC, we could pull down CALM with sufficiently high concentrations of VAMP7 (Figure 3f), indicating that CALM can interact directly with wild-type VAMP7 in spite of its longin domain.

Together, these data show that CALM is capable of trafficking VAMP4, but the dileucine motif is such a strong sorting signal normally that CALM is not required. CALM can also traffic VAMP7, even when it is full-length, but this ability becomes more apparent when the longin domain is deleted.

### Uncoupling the Two Functions of CALM

**Figure 4:**
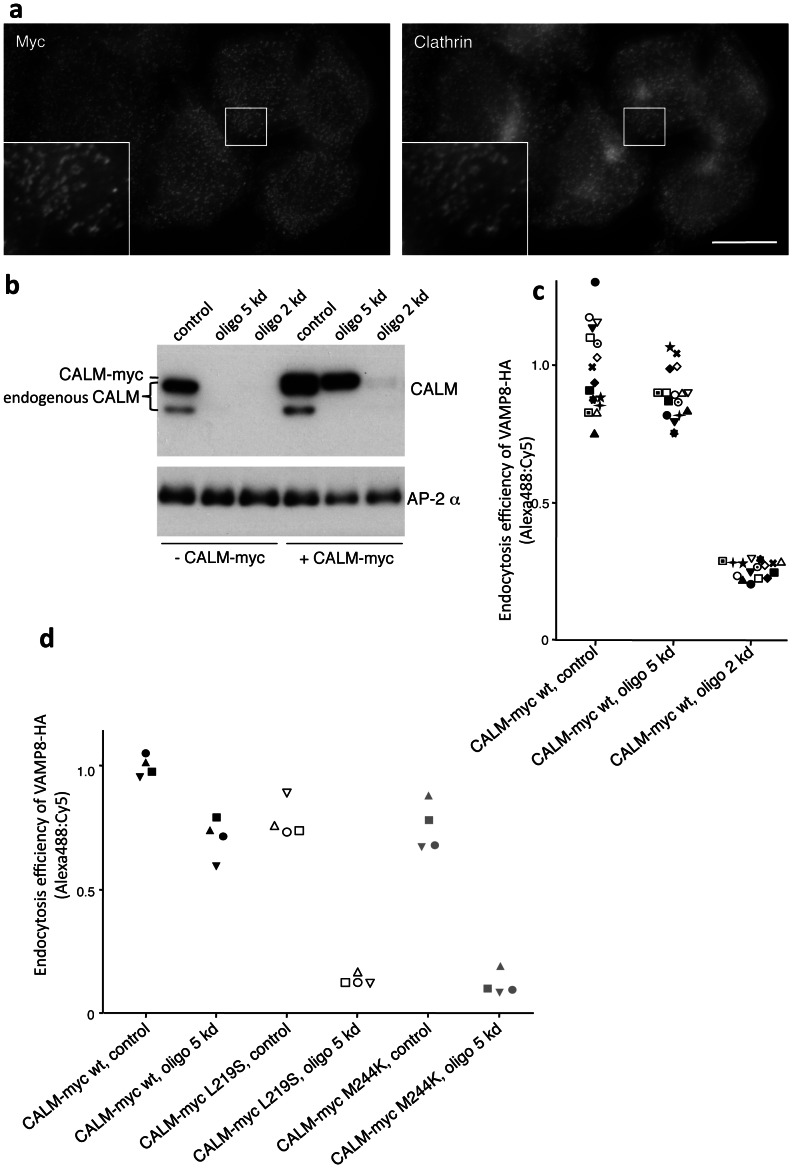
CALM knockdown/rescue system. a, Localization of myc-tagged siRNA-resistant CALM. The construct has a similar distribution to endogenous CALM, and colocalizes extensively with clathrin. Scale bar: 20 µm. b, Western blots of control and siRNA-treated cells. Two different siRNAs were used: oligo 5, which knocks down endogenous CALM but not the construct because it was made resistant to oligo 5, and oligo 2, which knocks down both endogenous CALM and the construct. The construct is expressed at similar levels to endogenous CALM. AP-2 α was included as a loading control. c, Endocytosis efficiency assay carried out on 16 cell lines coexpressing HA-tagged VAMP8 and myc-tagged siRNA-resistant CALM. Each symbol represents a different cell line. The values were normalized to 1 for the control. Oligo 2 inhibits endocytosis of VAMP8, but oligo 5 does not because the cells are rescued by the siRNA-resistant CALM construct. d, Endocytosis efficiency assay carried out on cells expressing CALM mutants. The L219S and M244K mutations abolish the interaction between CALM and VAMPs. Cells expressing these two mutants still endocytose VAMP8 when endogenous wild-type CALM is present, but when the endogenous CALM is knocked down, they fail to rescue the phenotype.

We recently developed a CALM rescue system to confirm the specificity of our knockdowns and to investigate the phenotypes of CALM mutants [6]. The system makes use of clonal cell lines coexpressing HA-tagged VAMP8 and myc-tagged CALM, with silent mutations engineered into the CALM cDNA to prevent it from annealing with one of our siRNAs, oligo 5. Immunofluorescence shows that the CALM constructs colocalize with clathrin, indicating that they are incorporated into clathrin-coated pits and vesicles (Figure 4a). Western blotting shows that knockdowns with oligo 5 deplete only endogenous CALM, leaving the tagged CALM unaffected, while knockdowns with another siRNA, oligo 2, deplete both endogenous and tagged CALM (Figure 4b). The blots also reveal that the tagged CALM is expressed at similar levels to endogenous CALM, which is important because overexpression of CALM has been shown to impair clathrin-mediated trafficking [23]. When the endocytosis efficiency of VAMP8 was assayed, multiple clonal cell lines were found to be resistant to oligo 5, but sensitive to oligo 2 (Figure 4c). In contrast, two mutants of CALM, CALM-myc L219S and CALM-myc M244K, which are known to abolish binding to the VAMPs [6] failed to rescue the VAMP8 endocytosis phenotype (Figure 4d).

**Figure 5:**
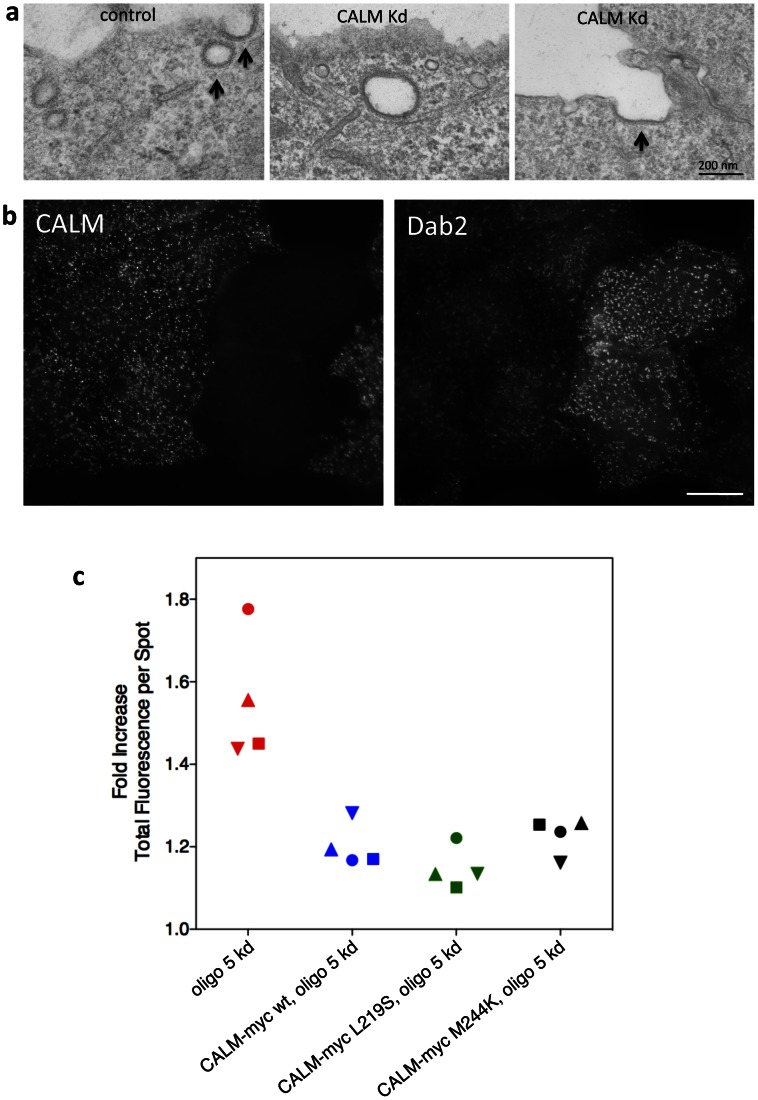
Morphology of coated pits in CALM-depleted cells. a, Electron micrographs of control and CALM-depleted cells, showing the enlarged, non-constricted pits that accumulate during a CALM knockdown. b, Mixture of control and CALM-depleted cells, double labeled for CALM and Dab2, another component of the endocytic clathrin coat. The brighter labeling of Dab2 in the CALM-depleted cells can be correlated with the enlarged coated pits seen by electron microscopy. c, Ability of wild-type and mutant CALM constructs to rescue the morphology of clathrin-coated pits. For each condition, Dab2 labeling was quantified by automated microscopy. CALM knockdown with oligo 5 caused a ∼60% increase in spot intensity. The three CALM constructs are resistant to oligo 5. Both the wild-type and the mutant constructs were able to rescue the morphological phenotype. This indicates that the change in coated pit morphology upon CALM knockdown is unrelated to the VAMP-sorting function of CALM.

To investigate the morphology of clathrin-coated pits in CALM-depleted cells, we first carried out electron microscopy. Figure 5a shows a similar phenotype to the one described by Meyerholz et al. [2]. Clathrin-coated pits in CALM-depleted cells tend to be larger than in control cells, and when observed in continuity with the plasma membrane, they often have a larger radius of curvature and wider neck (Figure 5a, arrow). To try to correlate the changes seen by electron microscopy with immunofluorescence images, we mixed together control and CALM-depleted cells and then double labeled for CALM and another endocytic CCV component, Dab2. Although an increase in the size of clathrin-coated pits would be difficult to see because of the limit of resolution of light microscopy, the pits are clearly brighter due to increased recruitment of machinery like Dab2 (Figure 5b). A similar observation was reported by Meyerholz et al. [2].

To quantify the effect of the CALM knockdown on Dab2 fluorescence, we made use of an assay that we recently developed for monitoring changes in CCP morphology by automated microscopy [21]. This assay enables us to analyse hundreds of cells in an objective manner, and to test and compare multiple clonal cell lines. When we quantified the intensity of Dab2-positive spots in cells treated with oligo 5, and compared it with the intensity in control cells, we found a ∼60% increase in the CALM-depleted cells (Figure 5c). Cells expressing oligo 5-resistant wild-type CALM-myc were largely rescued: Dab2-labeling increased by only 10–20% (Figure 5c). In addition, the two mutants, CALM-myc L219S and CALM-myc M244K, rescued the morphological phenotype just as well as wild-type CALM. This finding, together with our results showing that the same two mutants were unable to rescue VAMP8 endocytosis (Figure 4d), indicates that the role of CALM in controlling CCV size is independent of its role in sorting VAMPs.

## Discussion

In this study, we have made use of a versatile endocytosis efficiency assay, combined with a quantitative assay for clathrin-coated pit morphology, to investigate CALM function. We found that CALM can sort not only the brevin family VAMPs 2, 3, and 8, but also two other post-Golgi VAMPs, VAMP4 and VAMP7. In addition, we discovered that CALM’s role in VAMP sorting can be uncoupled from its role in controlling the size of coated pits.

Unlike VAMPs 2, 3, and 8, VAMP4 and VAMP7 do not absolutely require CALM for correct localization and trafficking because they have additional sorting signals. VAMP4 has a dileucine motif, which interacts particularly well with AP-1 in vitro [22], but which is likely to be recognized in vivo by AP-2 as well [24]. Thus, as long as the dileucine motif is present, VAMP4 remains intracellular even when CALM is depleted. However, when the dileucine is mutated, VAMP4 becomes CALM-dependent. This observation extends the work of Gordon et al. [15], who showed that both the dileucine motif and the SNARE domain need to be mutated for VAMP4 to accumulate on the plasma membrane, and it indicates that both AP complexes and CALM contribute to VAMP4 sorting.

Unlike the other post-Golgi VAMPs, VAMP7 has a longin domain, which binds back on the SNARE domain and causes the protein to adopt a closed conformation [25]. In this closed conformation, residues that participate in both four-helical bundle formation and CALM binding are inaccessible. The longin domain of VAMP7 has additional binding partners, including the δ subunit of the AP-3 adaptor complex, which facilitates the trafficking of VAMP7 to late endocytic organelles [26] [27], and the clathrin adaptor Hrb [10] [11], which facilitates VAMP7 endocytosis. Because Hrb binds to the same part of the longin domain that binds to the SNARE domain, it can only interact with VAMP7 when it is in its open conformation [10]. Thus, Hrb-mediated endocytosis of VAMP7 could potentially bring its SNARE partners into the endosome as well; or alternatively, VAMP7 could simultaneously interact with Hrb and CALM. Our finding that knocking down CALM has a subtle but highly reproducible effect on surface expression of wild-type VAMP7 suggests that Hrb and CALM may normally act together to facilitate the endocytosis of VAMP7. When the longin domain is deleted, VAMP7 remains predominantly intracellular, but now knocking down CALM causes a more dramatic increase on the plasma membrane.

The ability of CALM to sort VAMP7 may be relevant to many different types of eukaryotes. Hrb-related proteins are found only in animals, but nearly all eukaryotes have VAMP7-related proteins [28]. Indeed, in some organisms, such as Leishmania [29] and Arabidopsis [30], all of the post-Golgi VAMPs are most closely related to VAMP7 and have longin domains. This raises the question of how these VAMPs get retrieved from the plasma membrane. Interestingly, nearly all eukaryotes, including Leishmania and Arabidopsis, also have CALM homologues with ANTH domains (one protein [XP_001683520] in Leishmania; eight proteins in Arabidopsis [31] [32] [33]. Moreover, Wen et al. [34] have shown that in Dictyostelium, a CALM homologue is required for the correct sorting of VAMP7B (which has a longin domain), and they demonstrated that the two proteins interact in pulldown assays. Together, these observations suggest that in some organisms, CALM may be able to sort VAMP7-related proteins in spite of their longin domains, either by effectively competing with the longin domain for binding to the SNARE domain, or perhaps by harnessing another protein to occupy the longin domain. Thus, it is possible that CALM may have evolved as a universal adaptor for all post-Golgi VAMPs, not just those belonging to the brevin family, especially since the brevins appear to be restricted to unikonts (i.e., animals, fungi, and amoebae).

CALM displays similar recruitment dynamics to AP-2 [35], and a recent proteomics study indicates that CALM and AP-2 are of similar abundance in endocytic CCVs [36]. However, AP-2 depletion greatly reduces the number of clathrin-coated pits, and impairs the endocytosis of many cargo proteins [17] [37]. In contrast, CALM depletion does not appear to affect the number of coated pits, and most studies have found little or no effect on the rate of endocytosis of “standard” cargo proteins like the transferrin receptor in CALM-depleted cells [2] [6] [37], although reduced transferrin uptake has been observed in CALM knockout mice [38] [39]. Intriguingly, however, coated pits in CALM-depleted cells have been shown to be larger and more irregular than in controls; and in both C. elegans and Drosophila, mutations in CALM/AP180 homologues cause an increase in the size of synaptic vesicles, thought to be due to changes in the size of clathrin-coated vesicles, which are synaptic vesicle precursors [1] [12]. It is still unclear why loss of CALM might affect vesicle size, but we have demonstrated here that the ability of CALM to regulate clathrin-coated pit morphology is independent of its function to sort VAMPs. Thus, the role of CALM/AP180 in coated pit morphology likely relates to one or more of its other binding partners. These include PIP2, which (like VAMPs) binds to the N-terminal ANTH domain of CALM; and clathrin, AP-2, and other endocytic machinery like intersectin and Eps15, which bind to the C-terminal disordered domain. The neuronal-specific homologue of CALM, AP180, contains up to 12 clathrin-binding sites in its disordered domain [40], and thus might be able to control coated pit size by “tightening” the clathrin lattice; in contrast, CALM is thought to have fewer binding sites for clathrin [2], but it may play a similar role through its ability to bind to other coat components.

There has recently been a surge of interest in CALM because of its association with Alzheimer’s disease. So far, however, the molecular basis for this association is unclear, although it is thought to reflect the role of CALM in amyloid precursor protein trafficking, processing, and/or clearance [41]. The development of a CALM rescue system that can be used in different types of assays, to test different types of mutations, could potentially be used to learn more about the role of CALM in the pathogenesis of Alzheimer’s disease.

## References

[1] Nonet ML, Holgado AM, Brewer F, Serpe CJ, Norbeck BA, et al (1999) UNC-11, a Caenorhabditis elegans AP180 homologue, regulates the size and protein composition of synaptic vesicles. Mol Biol Cell 10: 2343–2360. 10397769 10.1091/mbc.10.7.2343 PMC25452

[2] Meyerholz A, Hinrichsen L, Groos S, Esk PC, Brandes G, et al (2005) Effect of clathrin assembly lymphoid myeloid leukemia protein depletion on clathrin coat formation. Traffic 6: 1225–1234. 16262731 10.1111/j.1600-0854.2005.00355.x

[3] Burston HE, Maldonado-Báez L, Davey M, Montpetit B, Schluter C, et al (2009) Regulators of yeast endocytosis identified by systematic quantitative analysis. J Cell Biol 185: 1097–1110. 19506040 10.1083/jcb.200811116 PMC2711619

[4] Harel A, Wu F, Mattson MP, Morris CM, Yao PJ (2008) Evidence for CALM in directing VAMP2 trafficking. Traffic 9: 417–429. 18182011 10.1111/j.1600-0854.2007.00694.x

[5] Koo SJ, Markovic S, Puchkov D, Mahrenholz CC, Beceren-Braun F, et al (2011) SNARE motif-mediated sorting of synaptobrevin by the endocytic adaptors clathrin assembly lymphoid myeloid leukemia (CALM) and AP180 at synapses. Proc Natl Acad Sci USA 108: 13540–13545. 21808019 10.1073/pnas.1107067108 PMC3158172

[6] Miller SE, Sahlender DA, Graham SC, Höning S, Robinson MS, et al (2011) The molecular basis for the endocytosis of small R-SNAREs by the clathrin adaptor CALM. Cell 147: 1118–1131. 22118466 10.1016/j.cell.2011.10.038 PMC3267021

[7] Wiederhold K, Kloepper TH, Walter AM, Stein A, Kienle N, et al (2010) A coiled coil trigger site is essential for rapid binding of synaptobrevin to the SNARE acceptor complex. J Biol Chem 285: 21549–21559. 20406821 10.1074/jbc.M110.105148 PMC2898431

[8] Kelly BT, Owen DJ (2011) Endocytic sorting of transmembrane protein cargo. Curr Opin Cell Biol 23: 404–412. 21450449 10.1016/j.ceb.2011.03.004

[9] Miller SE, Collins BM, McCoy AJ, Robinson MS, Owen DJ (2007) A SNARE-adaptor interaction is a new mode of cargo recognition in clathrin-coated vesicles. Nature 450: 570–574. 18033301 10.1038/nature06353

[10] Pryor PR, Jackson L, Gray SR, Edeling MA, Thompson A, et al (2008) Molecular basis for the sorting of the SNARE VAMP7 into endocytic clathrin-coated vesicles by the ArfGAP Hrb. Cell 134: 817–827. 18775314 10.1016/j.cell.2008.07.023 PMC2648964

[11] Chaineau M, Danglot L, Proux-Gillardeaux V, Galli T (2008) Role of HRB in clathrin-dependent endocytosis. J Biol Chem 283: 34365–34373. 18819912 10.1074/jbc.M804587200 PMC2662242

[12] Zhang B, Koh YH, Beckstead RB, Budnik V, Ganetzky B, et al (1998) Synaptic vesicle size and number are regulated by a clathrin adaptor protein required for endocytosis. Neuron 21: 1465–1475. 9883738 10.1016/s0896-6273(00)80664-9

[13] Harold D, Abraham R, Hollingworth P, Sims R, Gerrish A, et al (2009) Genome-wide association study identifies variants at CLU and PICALM associated with Alzheimer’s disease. Nat Genet 41: 1088–1093. 19734902 10.1038/ng.440 PMC2845877

[14] Rajendran L, Annaert W (2012) Membrane trafficking pathways in Alzheimer’s disease. Traffic 13: 759–770. 22269004 10.1111/j.1600-0854.2012.01332.x

[15] Gordon DE, Mirza M, Sahlender DA, Jakovleska J, Peden AA (2009) Coiled-coil interactions are required for post-Golgi R-SNARE trafficking. EMBO Rep 10: 851–856. 19557002 10.1038/embor.2009.96 PMC2726663

[16] Tiwari RK, Kusari J, Sen GC (1987) Functional equivalents of interferon-mediated signals needed for induction of an mRNA can be generated by double-stranded RNA and growth factors. EMBO J 6: 3373–3378. 2828026 10.1002/j.1460-2075.1987.tb02659.x PMC553793

[17] Motley A, Bright NA, Seaman MNJ, Robinson MS (2003) Clathrin-mediated endocytosis in AP-2-depleted cells. J Cell Biol 162: 909–918. 12952941 10.1083/jcb.200305145 PMC2172830

[18] Simpson F, Bright NA, West MA, Newman LS, Darnell RB, et al (1996) A novel adaptor-related protein complex. J Cell Biol 133: 749–760. 8666661 10.1083/jcb.133.4.749 PMC2120832

[19] Kozik P, Francis RW, Seaman MNJ, Robinson MS (2010) A screen for endocytic motifs. Traffic 11: 843–855. 20214754 10.1111/j.1600-0854.2010.01056.x PMC2882754

[20] Hirst J, Sahlender DA, Li S, Lubben NB, Borner GHH, et al (2008) Auxilin depletion causes self-assembly of clathrin into membraneless cages in vivo. Traffic 9: 1354–1371. 18489706 10.1111/j.1600-0854.2008.00764.x PMC2628426

[21] Kozik P, Hodson NA, Sahlender DA, Simecek N, Soromani C, et al (2013) A human genome-wide screen for regulators of clathrin-coated vesicle formation reveals an unexpected role for the V-ATPase. Nat Cell Biol 15: 50–60. 23263279 10.1038/ncb2652 PMC3588604

[22] Peden AA, Park GY, Scheller RH (2001) The di-leucine motif of vesicle-associated membrane protein 4 is required for its localization and AP-1 binding. J Biol Chem 276: 49183–49187. 11598115 10.1074/jbc.M106646200

[23] Tebar F, Bohlander SK, Sorkin A (1999) Clathrin assembly lymphoid myeloid leukemia (CALM) protein: localization in endocytic-coated pits, interactions with clathrin, and the impact of overexpression on clathrin-mediated traffic. Mol Biol Cell 10: 2687–2702. 10436022 10.1091/mbc.10.8.2687 PMC25500

[24] Kelly BT, McCoy AJ, Späte K, Miller SE, Evans PR, et al (2008) A structural explanation for the binding of endocytic dileucine motifs by the AP2 complex. Nature 456: 976–979. 19140243 10.1038/nature07422 PMC4340503

[25] Vivona S, Liu CW, Strop P, Rossi V, Filippini F, et al (2010) The longin SNARE VAMP7/TI-VAMP adopts a closed conformation. J Biol Chem 285: 17965–17973. 20378544 10.1074/jbc.M110.120972 PMC2878558

[26] Martinez-Arca S, Rudge R, Vacca M, Raposo G, Camonis J, et al (2003) A dual mechanism controlling the localization and function of exocytic v-SNAREs. Proc Natl Acad Sci USA 100: 9011–9016. 12853575 10.1073/pnas.1431910100 PMC166429

[27] Kent HM, Evans PR, Schäfer IB, Gray SR, Sanderson CM, et al (2012) Structural basis of the intracellular sorting of the SNARE VAMP7 by the AP3 adaptor complex. Dev Cell 22: 979–988. 22521722 10.1016/j.devcel.2012.01.018 PMC3549491

[28] Rossi V, Banfield DK, Vacca M, Dietrich LE, Ungermann C, et al (2004) Longins and their longin domains: regulated SNAREs and multifunctional SNARE regulators. Trends Biochem Sci 29: 682–688. 15544955 10.1016/j.tibs.2004.10.002

[29] Besteiro S, Coombs GH, Mottram JC (2006) The SNARE protein family of Leishmania major. BMC Genomics 7: 250. 17026746 10.1186/1471-2164-7-250 PMC1626469

[30] Uemura T, Ueda T, Ohniwa RL, Nakano A, Takeyasu K, et al (2004) Systematic analysis of SNARE molecules in Arabidopsis: dissection of the post-Golgi network in plant cells. Cell Struct Funct 29: 49–65. 15342965 10.1247/csf.29.49

[31] Barth M, Holstein SE (2004) Identification and functional characterization of Arabidopsis AP180, a binding partner of plant alphaC-adaptin. J Cell Sci 117: 2051–2062. 15054111 10.1242/jcs.01062

[32] Holstein SE, Oliviusson P (2005) Sequence analysis of Arabidopsis thaliana E/ANTH-domain-containing proteins: membrane tethers of the clathrin-dependent vesicle budding machinery. Protoplasma 226: 13–21. 16231097 10.1007/s00709-005-0105-7

[33] Field MC, Gabernet-Castello C, Dacks JB (2007) Reconstructing the evolution of the endocytic system: insights from genomics and molecular cell biology. Adv Exp Med Biol 607: 84–96. 17977461 10.1007/978-0-387-74021-8_7

[34] Wen Y, Stavrou I, Bersuker K, Brady RJ, De Lozanne A, et al (2009) AP180-mediated trafficking of Vamp7B limits homotypic fusion of Dictyostelium contractile vacuoles. Mol Biol Cell 20: 4278–4288. 19692567 10.1091/mbc.E09-03-0243 PMC2762145

[35] Taylor MJ, Perrais D, Merrifield CJ (2011) A high precision survey of the molecular dynamics of mammalian clathrin-mediated endocytosis. PLoS Biol e1000604. 10.1371/journal.pbio.1000604 PMC3062526 21445324

[36] Borner GHH, Antrobus R, Hirst J, Bhumbra GS, Kozik P, et al (2012) Multivariate proteomic profiling identifies novel accessory proteins of coated vesicles. J Cell Biol 197: 141–160. 22472443 10.1083/jcb.201111049 PMC3317806

[37] Huang F, Khvorova A, Marshall W, Sorkin A (2004) Analysis of clathrin-mediated endocytosis of epidermal growth factor receptor by RNA interference. J Biol Chem 279: 16657–16661. 14985334 10.1074/jbc.C400046200

[38] Suzuki M, Tanaka H, Tanimura A, Tanabe K, Oe N, et al.. (2012) The clathrin assembly protein PICALM is required for erythroid maturation and transferrin internalization in mice. PLoS One e31854. 10.1371/journal.pone.0031854 PMC3283694 22363754

[39] Scotland PB, Heath JL, Conway AE, Porter NB, Armstrong MB, et al.. (2012) The PICALM protein plays a key role in iron homeostasis and cell proliferation. PLoS One e44252. 10.1371/journal.pone.0044252 PMC3431333 22952941

[40] Zhuo Y, Ilangovan U, Schirf V, Demeler B, Sousa R, et al (2010) Dynamic interactions between clathrin and locally structured elements in a disordered protein mediate clathrin lattice assembly. J Mol Biol 404: 274–290. 20875424 10.1016/j.jmb.2010.09.044 PMC2981644

[41] Maritzen T, Koo SJ, Haucke V (2012) Turning CALM into excitement: AP180 and CALM in endocytosis and disease. Biol Cell 104: 588–602. 22639918 10.1111/boc.201200008

